# Ultrasound diagnosis and follow-up of a locked thumb metacarpophalangeal joint caused by radial sesamoid entrapment: a case report

**DOI:** 10.1186/s12891-020-03541-6

**Published:** 2020-07-31

**Authors:** Kyung-Sik Ahn, In Cheul Choi, Chang Ho Kang, Jong Woong Park

**Affiliations:** 1 Department of Radiology, Korea University Anam Hospital, Korea University College of Medicine, Seoul, South Korea; 2 Division of Hand Surgery & Reconstructive Microsurgery, Department of Orthopedic Surgery, Korea University Anam Hospital, Korea University College of Medicine, 73, Goryeodae-ro, Seongbuk-gu, Seoul, 02841 South Korea

**Keywords:** Locked thumb, Metacarpophalangeal joint, Sesamoid, Ultrasound

## Abstract

**Background:**

A locked thumb metacarpophalangeal joint is a rare condition that presents as restricted joint motions with mild hyperextension deformity, usually after a relatively minor hyperextension injury. Owing to the limitations of radiographs, computed tomography is a useful diagnostic imaging modality for assessing sesamoid displacement. However, despite its convenience, ultrasound findings of the locked thumb have rarely been reported. Here, we report a case of a locked thumb metacarpophalangeal joint diagnosed and followed-up using ultrasound.

**Case presentation:**

A 15-year-old boy with a locked thumb metacarpophalangeal joint presented to our hospital. On physical examination, the 1st metacarpophalangeal joint was found to be hyperextended, and active and passive flexions were not possible. While radiographs were inconclusive, ultrasound revealed radial sesamoid entrapment at the 1st metacarpophalangeal joint causing locking. After closed manual reduction, metacarpophalangeal motions recovered. Success of the reduction was also confirmable by ultrasound.

**Conclusions:**

Ultrasound can be a feasible modality to diagnose a locked thumb metacarpophalangeal joint and immediately judge the success or failure of the reduction.

## Background

Locking of the thumb metacarpophalangeal (MP) joint refers to a condition that causes a fixed state of the MP joint with a slight hyperextension deformity and motion restriction [1–5]. It can result from relatively minor hyperextension injuries. Although there are some controversies, an incarcerated volar plate, accessory collateral ligament, or entrapped radial sesamoid are considered to be possible causes of locking [1, 3]. Closed reduction can be attempted initially, but open reduction is often required, particularly when the presentation or diagnosis is delayed [4]. In the absence of clinical information, radial sesamoid movement or entrapment at the 1st MP joint is often overlooked on routine anteroposterior and lateral radiographs because of the bony overlap and variable degree of normal range of motion of the 1st MP joint [6]. Therefore, computed tomography (CT) is preferred for diagnosing a locked thumb to evaluate the location and status of the sesamoid [4, 5]. Although CT is an excellent modality to assess bony details, it causes radiation exposure and has a lower accessibility compared to ultrasound (US). US findings of a locked thumb have rarely been reported. Here, we report a case of a locked thumb MP joint in a patient with successful manual reduction in whom US findings before and after the reduction were useful to diagnose the disease and confirm the success of the treatment. We also discuss the feasibility of US and whether US can replace CT for diagnosing and managing the locked thumb MP joint.

## Case presentation

**Fig. 1:**
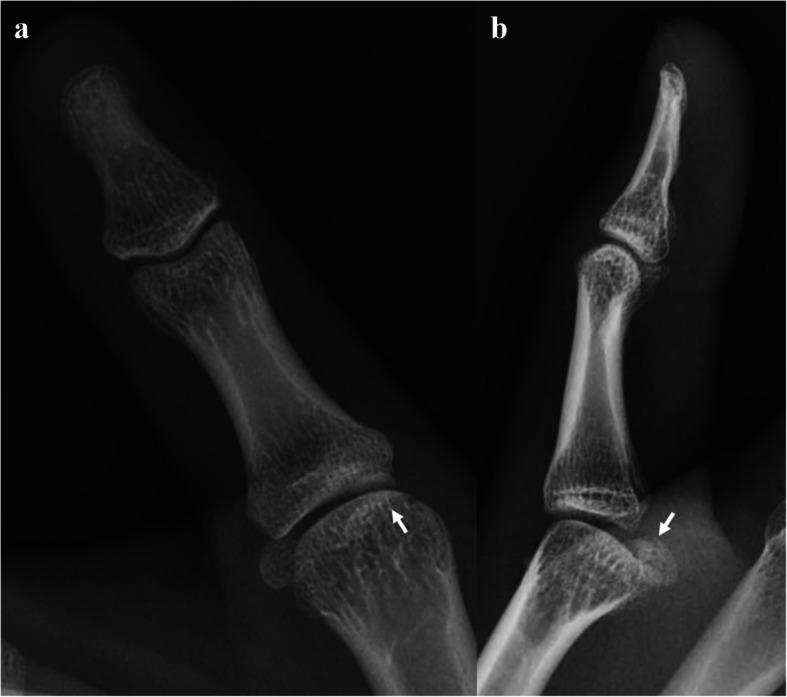
Anteroposterior (**a**) and lateral (**b**) radiographs of the right thumb. The lateral radiograph shows a hyperextension posture of the metacarpal joint at approximately 30°. The abnormal location of the radial sesamoid (arrow) is difficult to recognize on radiographs because of bony overlaps

**Fig. 2:**
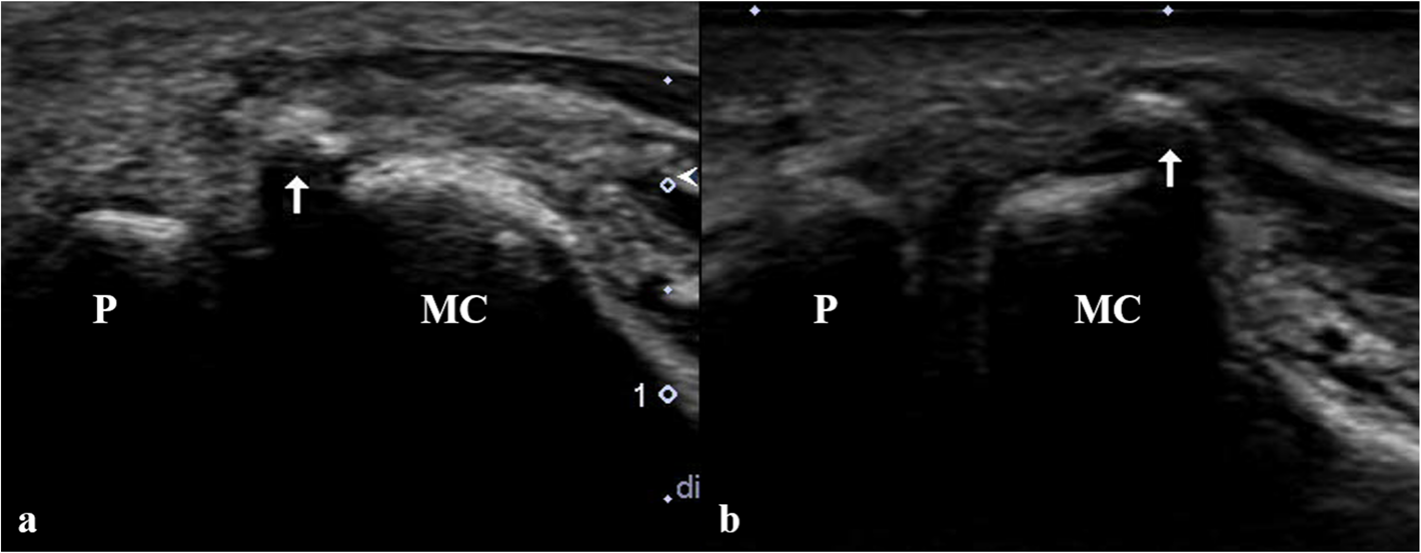
Longitudinal scan of ultrasound on volar surface of the 1st metacarpophalangeal joint shows distal displacement of the radial sesamoid bone (arrow) into the joint space (**a**). After reduction, the radial sesamoid has relocated to the radial condyle of the metacarpal head (**b**). P: proximal phalangeal base, MC: metacarpal head

**Fig. 3:**
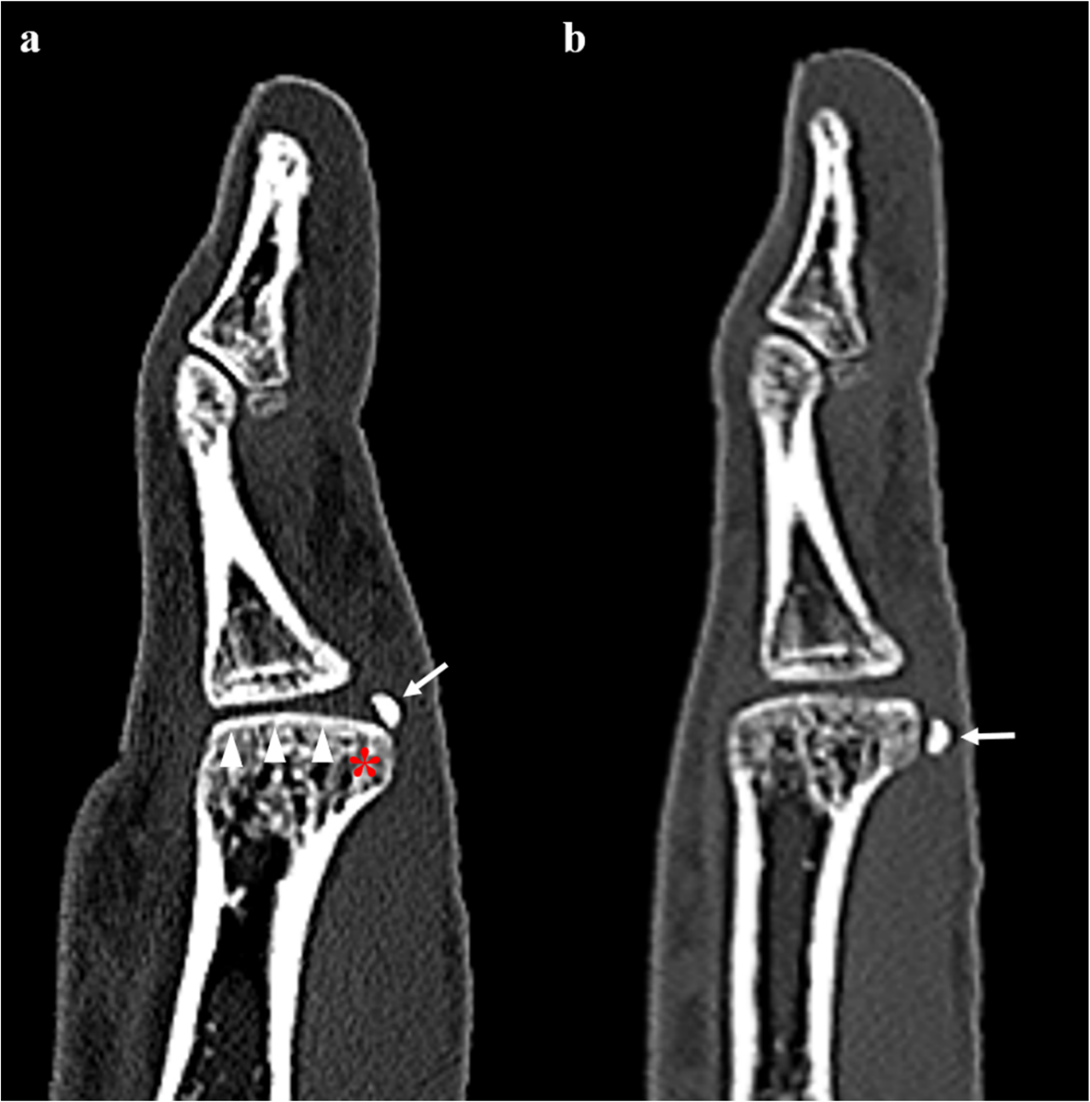
Sagittal reformation image of computed tomography (**a**) shows proximal displacement of the radial sesamoid (arrow). Note the non-round (flat) articular surface (arrow heads), prominence of the radial condyle of the metacarpal head (asterisk), and sharp proximal edge of the radial sesamoid. After reduction, the radial sesamoid (arrow) has relocated (**b**)

A 15-year-old boy complained of pain in the right thumb MP joint after a hyperextension injury while playing 2 weeks before. He was diagnosed with a ligament injury by an orthopedic surgeon at the first visit and referred to our hospital because his symptoms did not improve. On physical examination, the 1st MP joint was slightly hyperextended, and passive and active flexions were not possible. Radiographs showed a hyperextended posture at the 1st MP joint at approximately 30°. There were no definite abnormal findings on the first anteroposterior and lateral radiographs (Fig. 1). However, the clinical situation of the patient raised suspicion of a locked thumb MP joint, and we performed US. Distal displacement of the radial sesamoid was noted on US (Fig. 2a). Although limited, there were no conspicuous abnormalities in the volar plate or radial accessory collateral ligament on US. CT was also performed to ensure the diagnosis and evaluate the bony details. On CT, distal displacement of the radial sesamoid was visualized (Fig. 3a). In addition, a flat articular surface and prominent radial condyle of the metacarpal head were observed. We decided to attempt a closed manual reduction at the outpatient clinic under US guidance instead of a reduction under fluoroscopic guidance. After inducing local anesthesia with 1% lidocaine, closed manual reduction was attempted. After hyperextending the MP joint, continuous axial pressure was applied toward the metacarpal head, and subsequently, flexion of the MP joint was performed. The radial sesamoid was relocated with a snapping sound, and MP joint motion recovered. At that spot, we confirmed on US that the radial sesamoid returned to its place (Fig. 2b). A remo4vable thumb spica splint was applied for 2 weeks. Follow-up CT showed successful reduction of the radial sesamoid (Fig. 3b). During 3 months of follow-up, the patient did not have any recurrence.

## Discussion and conclusions

A locked thumb MP joint is rare. After mild hyperextension traumas of the MP joint, an inability to active or passive flexion is characteristic [3–5]. The cause of locking is unclear. Yamanaka et al. suggested that tearing of the proximal volar plate or accessory collateral ligament, which forms a constricting bundle over the distal metacarpal head, may cause locking [1]. Desai et al. reported entrapment of the sesamoid bone between the proximal phalangeal base and the metacarpal head, which causes locking [3]. Xiong et al. suggested that radial sesamoid entrapment under the flexor pollicis brevis and abductor pollicis brevis can block flexion, which results in locking [4]. Whether sesamoid entrapment is primary or secondary, it is an essential landmark for evaluating a locked thumb MP joint and can be a sentinel for adjacent soft tissue injuries. Inoue et al. reported that the bony morphology of the metacarpal head was a risk factor for locking [7]. The non-round shape of the articular surface and bony prominence of the radial condyle of the metacarpal head were observed with a high incidence among cases of locked thumb [1, 7]. The sharp proximal edge of the sesamoid is also suspected as a risk factor because it can easily damage and tear the membranous portion of the proximal volar plate, which results in distal displacement and locking [4].

Despite evident clinical symptoms, radiographs play a limited role in recognizing radial sesamoid displacement due to bony overlap. Most reports described distal and radial displacements of the sesamoid as an imaging finding of a locked thumb [1, 3]. Although the normal hyperextension posture can cause distal sliding of the sesamoid, prominent or asymmetric radial sesamoid displacement can be a sign of a locked thumb. US can easily identify the location of the radial sesamoid when radiographs are inconclusive or limited. To the best of our knowledge, US findings of a locked thumb MP joint have been reported only as a vignette [8]. Distal displacement of the radial sesamoid can be a finding of a locked thumb. Although we were unavailable at the time of diagnosis, a hockey stick transducer with a high frequency of over 12 MHz may be helpful for demonstrating soft tissue or cartilaginous lesions of the locked thumb, such as proximal volar plate tears, accessory collateral ligament abnormalities, and groove-like cartilage depression at the metacarpal head, all of which have been described in surgical cases [1–4]. Further, US has the advantages of high accessibility and real-time nature. An experienced physician can attempt closed reduction under US guidance and confirm the success or failure of the reduction immediately. CT is useful for evaluating bony details of the MP joint, including the shape of the articular surface, radial condyle of the metacarpal head, or edge shape of the sesamoid, which are known as risk factors for the locked thumb MP joint. The direct cortical abutment of the sesamoid and metacarpal head may indicate cartilage damage [4]. In our case, a flat metacarpal head and prominent radial condyle of the metacarpal head were observed on CT. We believe that US has the advantage of immediacy in diagnosis and treatment assessment over CT or magnetic resonance imaging (MRI) and is sufficiently replaceable. On the other hand, however, US has a limitation arising from operator dependency. Experienced physician who has knowledge of this disease and familiar with US can perform this kind of diagnosis. Further imaging study such as CT or MRI can be considered for irreducible cases, which require open reduction to evaluate the anatomic details of the metacarpal head and sesamoid to decide the surgical plan, such as shaving of the radial condyle prominence or excision of the sesamoid.

The treatment and clinical course of the locked thumb MP joint can vary. Some patients are treated completely without recurrence by manual reduction alone, but some fail to be treated with closed reduction and require open reduction. In surgery, excision of the incarcerated volar plate or accessory collateral ligament, shaving of the bony prominence of the radial condyle of the metacarpal head, or partial cutting of the flexor pollicis brevis can be performed to release locking and restore the motion [1–4]. Sesamoid excision is not recommended unless there is a cartilage depression on the metacarpal head or significant stripping of the sesamoid from the volar plate [3].

In conclusion, the locked thumb MP joint is a rare condition, but an accurate diagnosis is essential. For clinical information of a hyperextension injury at the MP joint and restricted passive motion, the radial sesamoid should be examined with a high index of suspicion. US can be considered before CT or MRI for prompt diagnosis and reduction assessment for a locked thumb MP joint.

## Data Availability

This is a case report of a single patient, to protect privacy and respect confidentiality; none of the raw data has been made available in any public repository. The original reports, imaging studies and outpatient clinic records are retained as per normal procedure within the medical records of our institution.

## Abbreviations

MP: Metacarpophalangeal
CT: Computed tomography
US: Ultrasound
MRI: Magnetic resonance imaging

## References

[1] Yamanaka K, Yoshida K, Inoue H, Inoue A, Miyagi T (1985). Locking of the metacarpophalangeal joint of the thumb. J Bone Joint Surg Am.

[2] Inoue G, Miura T (1988). Locked metacarpo-phalangeal joint of the thumb. J Hand Surg Br.

[3] Desai SS, Morgan WJ (1991). Locked thumb metacarpophalangeal joint caused by sesamoid entrapment. J Hand Surg Am..

[4] Xiong G, Gao Y, Guo S, Dai L, Liu K (2015). Pathoanatomy and treatment modifications of metacarpophalangeal joint locking of the thumb. J Hand Surg Eur Vol.

[5] Harada Y, Inui A, Mifune Y, Nishimoto H, Kokubu T, Hiroyuki F (2020). Treatment of locking of the Metacarpophalangeal joint of the thumb. J Hand Microsurg.

[6] Yoshida R, House HO, Patterson RM, Shah MA, Viegas SF (2003). Motion and morphology of the thumb metacarpophalangeal joint. J Hand Surg Am.

[7] Inoue S, Tsuboi K (2005). Locking of thumb MP joint -morphological risk factors. Japan Soc Surg Hand.

[8] Wu SY, Liu SY, Wei TS (2014). Early sonographic diagnosis and successful management of the sesamoid bone locking in the thumb. Am J Phys Med Rehabil.

